# Supplementary material for: HPV molecular detection from urine versus cervical samples: an alternative for HPV screening in indigenous populations
Source: PeerJ. 2021 Jun 17;9:e11564. doi: 10.7717/peerj.11564 (PMC8214846; doi:10.7717/peerj.11564)
Supplement: Supplemental Information 3 [file peerj-09-11564-s003.docx]

**Codebook to convert numbers to their respective factors:**

| **Variable name** | **number** | **Factor** |
| --- | --- | --- |
| BMI_cat | 0 | Undetermined |
|  | 1 | Under weight |
|  | 2 | Normal weight |
|  | 3 | Overweight |
|  | 4 | Obesity grade 1 |
|  | 5 | Obseity grade 2 |
|  | 6 | Obesity grade 3 |
| Civil_status | 0 | No data |
|  | 1 | Single |
|  | 2 | Spouse |
|  | 3 | Married |
|  | 4 | Divorcee |
|  | 5 | Widow |
| Partners_cond | 0 | 1 |
|  | 1 | 2-3 |
|  | 2 | 4 or more |
| coparas | 0 | None |
|  | 1 | 1-2 |
|  | 2 | 3 or more |
|  | 3 | No data |
| Alcohol | 0 | No |
|  | 1 | Yes |
| Exercise | 0 | No |
|  | 1 | Yes |
| Papanicolaou | 0 | No |
|  | 1 | Yes |
| Diabetes | 0 | No |
|  | 1 | Yes |
| Hypertension | 0 | No |
|  | 1 | Yes |
| ph | 0 | Undetermined |
|  | 1 | 4 or minus |
|  | 2 | 5-6 |
|  | 3 | 7 or more |
| KOH | 0 | Negative |
|  | 1 | Positive |
| Cytological_Diagnosis | 0 | Non-lesion |
|  | 1 | LSIL |
| Class_HPVc | 0 | Negative |
|  | 1 | HPV-LR |
|  | 2 | HPV-HR |
|  | 3 | IM_HPV_LR/HR |
|  | 4 | IM_HPV_HR |
|  | 5 | HPV_X |

| **Variable name** | **number** | **Factor** |
| --- | --- | --- |
| Viral_infection_type | 0 | No infection |
|  | 1 | One only virus |
|  | 2 | Multiple infection |
|  | 3 | No made |
|  | 4 | Invalid |
| Sediment | 0 | Scarce |
|  | 1 | Abundant |
| Density | 0 | 1.000 |
|  | 1 | 1.005 |
|  | 2 | 1.010 |
|  | 3 | 1.015 |
|  | 4 | 1.020 |
|  | 5 | 1.025 |
|  | 6 | 1.030 |
| Nitrites | 0 | Negative |
|  | 1 | Positive |
| Proteins | 0 | Negative |
|  | 1 | 30 mg/dl |
|  | 2 | 100 mg/dl |
|  | 3 | 500 mg/dl |
| CETONA | 0 | Negative |
|  | 1 | 1+ |
|  | 2 | 2+ |
|  | 3 | 3+ |
| Urin_Density_Cat | 0 | 1.01-1.02 |
|  | 1 | < 1.01 |
|  | 2 | > 1.02 |
| Leucos_cat | 0 | Negative |
|  | 1 | Positive |
| Protein_cat | 0 | Negative |
|  | 1 | Positive |
| Gluc_cat | 0 | Negative |
|  | 1 | Positive |
| EryHemo_Cat | 0 | Negative |
|  | 1 | Positive |
