# Supplementary figures and images for: HPV molecular detection from urine versus cervical samples: an alternative for HPV screening in indigenous populations
Source: PeerJ. 2021 Jun 17;9:e11564. doi: 10.7717/peerj.11564 (PMC8214846; doi:10.7717/peerj.11564)

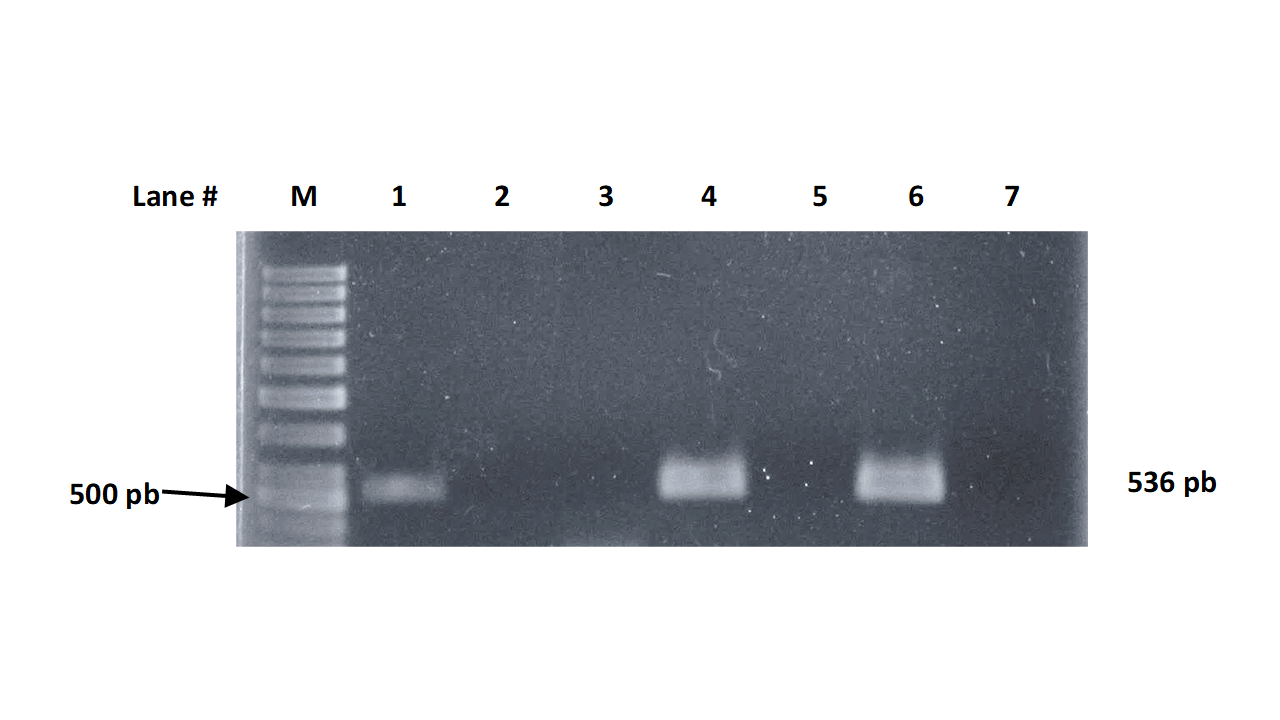

Supplement: Supplemental Information 4 — Total DNA was isolated from urine samples of indigenous women. M = size markers for double strand DNA, as indicated. DNA-fragments indicative for β-globin fragment (536 bp) as indicator of DNA integrity (lanes 1 and 4). Samples not amplification in lanes 2, 3 and 5. Lane 6 is a positive control (a clinic sample with a previous detection of viral DNA) and, finally, lane 7 show the negative control, in which no DNA was added. [file peerj-09-11564-s004.png]
