# Supplementary material for: HPV molecular detection from urine versus cervical samples: an alternative for HPV screening in indigenous populations
Source: PeerJ. 2021 Jun 17;9:e11564. doi: 10.7717/peerj.11564 (PMC8214846; doi:10.7717/peerj.11564)
Supplement: Supplemental Information 5 [file peerj-09-11564-s005.docx]

Table S1. HPV genotypes detected from cervical and urine samples.

| **HPV** | **Cervix**  **n (%)** | **Urine**  **n (%)** | **Agreement** |
| --- | --- | --- | --- |
| **HR-HPV** | | | |
| 16 | 4 (3.70) | 17 (15.74) | 2 |
| 18 | 2 (1.85) | 1 (0.92) | 0 |
| 31 | 7 (6.48) | 6 (5.56) | 3 |
| 33 | 0 | 4 (3.70) | 0 |
| 35 | 3 (2.78) | 4 (3.70) | 1 |
| 39 | 6 (5.56) | 21 (19.44) | 4 |
| 45 | 2 (1.85) | 2 (1.85) | 0 |
| 51 | 7 (6.48) | 15 (13.89) | 2 |
| 52 | 10 (9.26) | 16 (14.81) | 8 |
| 56 | 5 (4.63) | 5 (4.63) | 3 |
| 58 | 9 (8.33) | 12 (11.11) | 3 |
| 59 | 9 (8.33) | 10 (9.26) | 3 |
| 66 | 4 (3.70) | 7 (6.48) | 2 |
| 68 | 5 (4.63) | 7 (6.48) | 2 |
| 73 | 0 | 1 (0.92) | 0 |
| 53 | 4 (3.70) | 6 (5.56) | 1 |
| **LR-HPV** | | | |
| 6 | 1 (0.92) | 11 (10.2) | 0 |
| 11 | 0 | 2 (1.85) | 0 |
| 40 | 1 (0.92) | 0 | 0 |
| 43 | 1 (0.92) | 2 (1.85) | 1 |
| 44 | 2 (1.85) | 3 (2.78) | 1 |
| 54 | 4 (3.70) | 2 (1.85) | 1 |
| 61 | 3 (2.78) | 1 (0.92) | 0 |
| 62 | 6 (5.56) | 2 (1.85) | 1 |
| 70 | 2 (1.85) | 8 (7.41) | 2 |
| 82 | 3 (2.78) | 4 (3.70) | 2 |
| 89 | 1 (0.92) | 0 | 0 |
| **Undetermined VPH** | | | |
| VPH-X | 11 (10.18) | 10 (9.26) | 0 |
|  | | | |
