# Supplementary material for: HPV molecular detection from urine versus cervical samples: an alternative for HPV screening in indigenous populations
Source: PeerJ. 2021 Jun 17;9:e11564. doi: 10.7717/peerj.11564 (PMC8214846; doi:10.7717/peerj.11564)
Supplement: Supplemental Information 6 — *Fisher exact test [file peerj-09-11564-s006.docx]

| Table S2. Physicochemical characteristics of urine and its relationship with the type of HPV infection in cervix | | | | | | | |  |
| --- | --- | --- | --- | --- | --- | --- | --- | --- |
|  | HPV infection | | | | | |  | |
|  | Negative | LR-HPV | HR-HPV | MI  LR/HR-HPV | MI  HR-HPV | X-HPV | *p* value* | |
|  |  |  |  |  |  |  |  | |
| **Sediment** |  |  |  |  |  |  |  | |
| Scarce | 17 (53.13) | 2 (75.00) | 11 (28.95) | 3 (53.33) | 5 (38.46) | 3 (27.27) | 0.3 | |
| Abundant | 15 (46.87) | 1 (25.00) | 27 (71.05) | 5 (46.67) | 8 (61.54) | 8 (72.73) |  | |
| **Density** |  |  |  |  |  |  |  | |
| 1.01-1.02 | 24 (75.00) | 2 (66.67) | 27 (71.05) | 5 (53.33) | 11 (84.62) | 10 (90.91) |  | |
| <1.01 | 7 (21.88) | 1 (33.33) | 7 (18.42) | 2 (33.33) | 2 (15.38) | 0 (0) | 0.6 | |
| >1.02 | 1 (3.12) | 0 (0) | 4 (10.53) | 1 (13.34) | 0 (0) | 1 (9.09) |  | |
| **PH** |  |  |  |  |  |  |  | |
| 5 | 18 (56.25) | 1 (33.33) | 24 (63.16) | 5 (40.00) | 8 (61.54) | 6 (60) |  | |
| 6 | 9 (28.12) | 2 (66.67) | 12 (31.58) | 0 (33.33) | 3 (23.08) | 2 (20.00) | 0.23 | |
| 7 | 5 (15.63) | 0 (0) | 2 (5.26) | 3 (26.67) | 2 (15.38) | 3 (20.00) |  | |
| **Leucocytes** |  |  |  |  |  |  |  | |
| Negative | 28 (87.50) | 3 (100.00) | 31 (81.58) | 6 (80.00) | 9 (69.23) | 9 (81.82) | 0.69 | |
| Positive | 4 (12.50) | 0 (0) | 7 (18.42) | 2 (20.00) | 4 (30.77) | 2 (18.18) |  | |
| **Proteins** |  |  |  |  |  |  |  | |
| Negative | 22 (68.75) | 2 (66.67) | 21 (55.26) | 7 (73.33) | 10 (76.92) | 5 (45.45) | 0.3 | |
| Positive | 10 (31.25) | 1 (33.33) | 17 (44.74) | 1 (26.67) | 3 (23.08) | 6 (54.55) |  | |
| **Glucose** |  |  |  |  |  |  |  | |
| Negative | 25 (78.12) | 3 (100.00) | 32 (84.21) | 7 (80.00) | 10 (76.92) | 9 (81.82) | 0.97 | |
| Positive | 7 (21.88) | 0 (0) | 6 (15.89) | 1 (20.00) | 3 (23.08) | 2 (18.18) |  | |
| **Erythrocytes** |  |  |  |  |  |  |  | |
| Negative | 29 (90.63) | 3 (100.00) | 32 (84.21) | 6 (75.00) | 11 (84.62) | 11 (100.00) | 0.56 | |
| Positive | 3 (9.37) | 0 (0) | 6 (15.79) | 2 (25.00) | 2 (15.38) | 0 (0) |  | |
| *Fisher exact test | | | | | | | |  |
