# Supplementary material for: HPV molecular detection from urine versus cervical samples: an alternative for HPV screening in indigenous populations
Source: PeerJ. 2021 Jun 17;9:e11564. doi: 10.7717/peerj.11564 (PMC8214846; doi:10.7717/peerj.11564)
Supplement: Supplemental Information 7 — aX2 test, b Fisher exact test [file peerj-09-11564-s007.docx]

| Table S3. Physicochemical characteristics of urine and its relationship with cytological diagnosis | | | | |
| --- | --- | --- | --- | --- |
|  | cytological diagnosis | | |  |
|  | NSIL | LSIL | Total | *p* value |
|  | n (%) | n (%) | n (%) |  |
|  |  |  |  |  |
| **Sediment** |  |  |  |  |
| Scarce | 15 (46.88) | 26 (35.62) | 41 (39.05) | 0.27^a^ |
| Abundant | 17 (53.12) | 47 (64.38) | 64 (61.95) |  |
| **Density** |  |  |  |  |
| 1.01-1.02 | 24 (75.00) | 55 (75.34) | 79 (75.24) |  |
| <1.01 | 7 (21.88) | 12 (16.44) | 19 (18.09) | 0.56^b^ |
| >1.02 | 1 (3.12) | 6 (8.22) | 7 (6.67) |  |
| **PH** |  |  |  |  |
| 5 | 17 (53.12) | 45 (61.64) | 62 (59.05) |  |
| 6 | 10 (31.25) | 18 (24.66) | 28 (26.67) | 0.70^b^ |
| 7 | 5 (15.63) | 10 (13.70) | 15 (14.28) |  |
| **Leucocytes** |  |  |  |  |
| Negative | 27 (84.38) | 59 (80.82) | 86 (81.90) | 0.60^b^ |
| Positive | 5 (15.62) | 14 (19.18) | 19 (18.10) |  |
| **Proteins** |  |  |  |  |
| Negative | 22 (68.75) | 45 (61.64) | 67 (63.81) | 0.48^a^ |
| Positive | 10 (31.25) | 28 (38.36) | 38 (36.19) |  |
| **Glucose** |  |  |  |  |
| Negative | 24 (75.00) | 62 (84.93) | 86 (81.90) | 0.22^a^ |
| Positive | 8 (25.00) | 11 (15.07) | 19 (18.10) |  |
| **Erytrocites** |  |  |  |  |
| Negative | 29 (90.63) | 63 (86.30) | 92 (87.62) | 0.53^b^ |
| Positive | 3 (9.37) | 10 (13.70) | 13 (12.38) |  |
| ^a^ X^2^ test, ^b^ Fisher exact test | | | | |
